# Surgical Strategy for Hepatocellular Carcinoma Patients with Portal/Hepatic Vein Tumor Thrombosis

**DOI:** 10.1371/journal.pone.0130021

**Published:** 2015-06-15

**Authors:** Li Shaohua, Wang Qiaoxuan, Sun Peng, Li Qing, Yang Zhongyuan, Shi Ming, Wei Wei, Guo Rongping

**Affiliations:** 1 Department of Hepatobiliary Surgery, Cancer Center of Sun Yat-Sen University, Guangzhou, China; 2 State Key Laboratory of Oncology in South China, Collaborative Innovation Center for Cancer Medicine, Cancer Center of Sun Yat-Sen University, Guangzhou, China; 3 Department of Radiation Oncology, Cancer Center of Sun Yat-Sen University, Guangzhou, China; 4 Department of Anesthesia, Cancer Center of Sun Yat-Sen University, Guangzhou, China; 5 Department of Ultrasound, Cancer Center of Sun Yat-Sen University, Guangzhou, China; 6 Department of Head and Neck Surgery, Cancer Center of Sun Yat-Sen University, Guangzhou, China; Nanjing, CHINA

## Abstract

**Background:**

Portal/hepatic vein tumor thrombosis (PVTT/HVTT) in hepatocellular carcinoma (HCC) is a sign of advanced stage disease and is associated with poor prognosis. This study investigated the surgical outcomes of patients with HCC and PVTT/HVTT to determine the most appropriate surgical treatment strategy for these patients.

**Materials and Methods:**

The study population included 77 HCC patients from January 2004 to June 2009 who underwent hepatectomy in our department and were diagnosed with PVTT/HVTT based on pathological examination. The patients were divided into two groups: in group 1, PVTT/HVTT was located in the hepatic resection area and removed with the tumor en bloc (38 cases); in group 2, PVTT/HVTT was beyond the resection line and removed by suction or thrombectomy (39 cases). Concerning the factor of surgical margins, the patients were further divided into four subgroups: group 1A: patients in group 1 with surgical margins ≤1 cm (28 cases); group 1B: patients in group 1 with surgical margins >1 cm (9 cases); group 2A: patients in group 2 with surgical margins ≤1 cm (28 cases); and group 2B: patients in group 2 with surgical margins >1 cm (9 cases).

**Results:**

Most of the characteristics of groups 1 and 2 were similar. Patients in group 2 had significantly higher median blood loss (p=0.002) and higher blood transfusion rate (p=0.002) during the operation, which were not considered prognostic factors (p=0.323 and 0.571, respectively). The median overall survival (OS) duration in group 1 was significantly longer than that in group 2 (14.3 vs. 10.4 months, p=0.047). The median OS durations in groups 1A, 1B, 2A, and 2B were 14.3, 42.7, 7.5, and 18.0 months, respectively, which were significantly different(p=0.018).

**Conclusions:**

When PVTT/HVTT is located in the hepatic resection area and removed with the tumor en bloc, the median OS duration is longer. Based on this finding, widening the surgical margins when technically possible may increase OS.

## Introduction

Portal/hepatic vein tumor thrombosis (PVTT/HVTT) is a common complication of hepatocellular carcinoma (HCC) [1,2] and is widely accepted as a sign of advanced stage[3,4]. In some patients, PVTT/HVTT may be the initial sign of an undetected HCC [5] or may be observed after treatment, including ablation or chemoembolization, as a first indicator of recurrence [3]. Reports from Japan and China indicated that 808 of 5130 patients (15.8%) and 441 of 4954 patients (8.9%), respectively, who underwent hepatic resection developed macroscopic portal venous invasion [6,7].

The presence of PVTT/HVTT with HCC determines the choice of therapeutic strategies. PVTT/HVTT frequently leads to intrahepatic or distant metastasis with poor prognosis [8]. The median survival of untreated HCC with PVTT/HVTT has been reported to be 2.7 months, whereas the survival in those without PVTT/HVTT has been reported to be 24.4 months [9,10]. To date, the management of HCC with PVTT/HVTT remains complicated and controversial. Although many researchers believed that the presence of PVTT/HVTT in HCC is an absolute contraindication for liver transplantation, resection, and percutaneous ablation techniques and a relative contraindication for transcatheter hepatic arterial chemoembolization (TACE) [9,11,12], accumulated evidence has shown that hepatectomy with thrombectomy or en bloc resection can improve the survival of HCC patients with PVTT/HVTT [13–17].

The potential benefits of surgery in patients with HCC and PVTT/HVTT have not been thoroughly evaluated. This study aimed to investigate the surgical outcomes of patients with HCC and PVTT/HVTT and determine the most appropriate surgical treatment strategy for these patients.

## Patients and Methods

### Patients

The study population included 77 HCC patients who underwent hepatectomy in our department and were diagnosed with PVTT/HVTT based on pathological examination between January 2004 and June 2009. During the same period, there were 896 patients who had hepatocellular carcinoma and were treated by hepatectomy in our department.

Patients were excluded from analysis if they had extrahepatic disease or thrombus extending to the level of the superior mesenteric vein or if the received any treatments before surgery.

### Preoperative diagnosis and management

A preoperative diagnosis was verified by color Doppler ultrasonography (CDUS), tri-phase contrast enhanced helical computed tomography (CT), or contrast enhanced magnetic resonance (MR). There were 40 (51.95%) cases who had radiological evidence of tumor thrombosis preoperatively.

Liver function was evaluated based on the Child-Pugh classification system[18] and/or the indocyanine green (ICG) clearance test performed before surgery.

### Surgical treatment

The selection criteria for the operative procedure depended on tumor location and extent, liver function, and future liver remnant volume. Hepatectomy was defined as major if three or more Couinaud segments were resected and minor if fewer than three segments were resected [19]. Intraoperative ultrasonography was routinely performed to determine the size and location of the tumor and thrombus, the relationship between the tumor and the vascular system, and any undetected tumor in the remnant liver. The diagnosis of HCC and PVTT/HVTT was confirmed by histopathological examination of the resected specimens. There are 3 of 77 (3.90%) cases with hepatic vein tumor thrombosis alone and 4 of 77 (5.19%) cases with portal vein tumor thrombosis and hepatic vein tumor thrombosis simultaneously.

According to the location and extent of PVTT/HVTT, the relationship between thrombus, tumor, and resection line, and the surgical strategy, 77 patients were divided into two groups: group 1: PVTT/HVTT was located in the hepatic resection area and removed with the tumor en bloc (38 cases); and group 2: PVTT/HVTT extended beyond the resection line and was removed by suction or thrombectomy (39 cases).

Concerning the factor of surgical margin, we further divided the two groups into four subgroups: group 1A: PVTT/HVTT was located in the hepatic resection area and removed with the tumor en bloc with surgical margins≤1 cm (28 cases); group 1B: PVTT/HVTT was located in the hepatic resection area and removed with the tumor en bloc with surgical margins>1 cm (9 cases);group 2A: PVTT/HVTT extended beyond the resection line and was removed by suction or thrombectomy with surgical margins≤1 cm (28 cases); and group 2B: PVTT/HVTT extended beyond the resection line and was removed by suction or thrombectomy with surgical margins>1 cm (9 cases). The surgical margins of three patients were unknown.

### Postoperative care and follow-up

Operative mortality was defined as death within 90 days after the operation. Operative complication was defined as any deviation from the normal course of recovery with the need for pharmacological, surgical, radiological, or endoscopic intervention.

All patients were followed up 1 month after the operation with enhanced CT of the chest and upper abdomen, liver function tests, and serum α-fetoprotein (AFP) examination. Thereafter, they were followed up every 2–3 months using radiology (enhanced CT of the chest and upper abdomen or combined CDUS and chest X-ray) and serum examination in the first postoperative year. After the first year, all patients were followed up every 3–6 months with CDUS, chest X-ray, and serum tests. Abdominal enhanced CT, abdominal enhanced MR, and/or contrast-enhanced ultrasonography (CEUS) were performed when intrahepatic recurrence was suspected, and thoracic enhanced CT, whole-body bone scintigraphy, or/and other relevant radiologic examination were performed when extrahepatic recurrence was suspected.

Patients with recurrence were treated with the following based on their liver function and the pattern of recurrence: TACE, transarterial infusion (TAI), radiofrequency ablation (RFA), percutaneous microwave tumor coagulation therapy (PMCT), hepatectomy, percutaneous ethanol injection therapy (PEI), sealed source radiotherapy, systemic chemotherapy, sorafenib, cytokine-induced killer (CIK) cells therapy, and/or supportive care.

### Ethics Statement

This research was approved by the institutional review board(IRB) of Sun Yat-sen University Cancer Center, and written informed consent was obtained from each patient.

### Statistical analysis

Comparisons between categorical variables were performed using Pearson’s χ^2^ test or Fisher’s exact test where appropriate. Continuous variables were compared using Student's t-test (when values were normally distributed) or the Mann-Whitney test (when values had a distribution that departed significantly from normal). The survival analysis was calculated using the Kaplan-Meier method and compared using the log-rank test. A multivariate analysis using Cox’s proportional hazard model was performed to evaluate the prognostic factors. A value of *p*<0.05 was considered statistically significant. All data were analyzed using SPSS statistical software for Windows (ver. 18.0; SPSS Inc., Chicago, IL).

All continuous variable data are expressed as the mean ±standard error (when values were normally distributed) or the median (range) (when values had a distribution that departed significantly from normal). All data regarding categorical variables are shown as n (proportion).

## Results

### Clinicopathological characteristics

The characteristics of the 77 HCC patients with PVTT/HVTT are summarized in Table 1. Most of the characteristics of the two groups were similar. Patients in group 2 had significantly higher median blood loss (*p* = 0.002) and blood transfusion rates (*p* = 0.002) during the operation.

**Table 1:** Clinicopathological characteristics of the 77 HCC patients with PVTT/HVTT.

	Group 1 (n = 38)	Group 2 (n = 39)	*p* value
Age (years)	49.3±1.5	47.9±2.2	0.609
Gender			0.571
Male	37 (97.4%)	37 (94.9%)	
Female	1 (2.6%)	2 (5.1%)	
HBsAg status			0.556
Negative	2 (5.3%)	1 (2.6%)	
Positive	35 (92.1%)	36 (92.3%)	
Unknown	1 (2.6%)	2 (5.1%)	
Preoperative HBV-DNA			0.662
<1×103	10 (26.3%)	12 (30.8%)	
≥1×103	20 (52.6%)	19 (48.7%)	
Unknown	8 (21.1%)	8 (20.5%)	
Preoperative AFP level			0.935
≤400 ng/ml	13 (34.2%)	13 (33.3%)	
>400 ng/ml	25 (65.8%)	26 (66.7%)	
Preoperative ALT level (U/L)	43(10~713)	40.5(14~208)	0.433
Preoperative Hgb level (g/L)	148.54±3.27	145.74±3.08	0.534
Preoperative PLT level (109/L)	197.7±16.7	183.4±11.0	0.472
Preoperative Child-Pugh score			0.651
Child A (5)	11 (28.9%)	15 (38.5%)	
Child A (6)	18 (47.4%)	17 (43.6%)	
Child B (7)	5 (13.2%)	3 (7.7%)	
Child B (8)	2 (5.3%)	1 (2.6%)	
Child B (9)	1 (2.6%)	0 (0.0%)	
Unknown	1 (2.6%)	3 (7.7%)	
Preoperative ICGR15 (%)	5.58±0.96	4.46±0.68	0.342
Number of tumors			0.424
Solitary	21 (55.3%)	18 (46.2%)	
Multiple	17 (44.7%)	21 (53.8%)	
Maximum diameter of tumor (cm)	9.91±0.71	10.08±0.71	0.866
Uni/Bilobular disease			0.249
Unilobular disease	36 (94.7%)	34 (87.2%)	
Bilobular disease	2 (5.3%)	5 (12.8%)	
Adjacent organ invasion			0.883
Negative	24 (63.2%)	24 (61.5%)	
Positive	14 (36.8%)	15 (38.5%)	
Operative procedure			0.289
Minor	24 (63.2%)	29 (74.4%)	
Major	14 (36.8%)	10 (25.6)	
Total occlusion time of the hepatic inflow (min)	15.95±2.11	20.64±1.86	0.098
Total operative time (min)	176.2±11.2	186.4±9.8	0.492
Blood loss (ml)	350 (100~4300)	600 (100~2000)	0.002
Blood transfusion (ml)	0±2300	200±1700	0.002
Surgical margin			1.000
≤1 cm	28 (73.7%)	28 (71.8%)	
>1 cm	9 (23.7%)	9 (23.1%)	
Unknown	1 (2.6%)	2 (5.1%)	
Histologic grade of tumor cells^a^			0.527
I-II	12 (31.6%)	15 (38.5%)	
III-IV	26 (68.4%)	24 (61.5%)	
Postoperative complication			0.817
Negative	31 (81.6%)	31 (79.5%)	
Positive	7 (18.4%)	8 (20.5%)	
Postoperative hospital stay (days)	11(8~28)	11(8~83)	0.762

HBsAg: surface antigen of the hepatitis B virus; ALT: alanine transaminase; Hgb: hemoglobin; PLT: platelet; ICGR15: indocyanine green retention rate at 15 min

^a^Histologic grade of HCC was assessed according to the Edmondson-Steiner grade system [45].

### Perioperative outcomes

The overall postoperative complications were not significantly different between the two groups (*p* = 0.817) and are shown in Table 2.

**Table 2:** Postoperative complications of the 77 HCC patients with PVTT/HVTT.

Type of complication	Group 1 (n = 38)	Group 2 (n = 39)
Pleural effusion	2	5
Severe ascites	3	2
Jaundice	2	2
Edema of lower limbs and/or scrotum	1	4
Liver failure	1	0
Wound infection	1	0
Intra-abdominal abscess	1	1
Intra-thoracic abscess	0	1
Palpitation	1	0
Sepsis	0	1
Biliary fistula	0	1

Some patients had more than one complication

### Survival analysis

**Fig 1:**
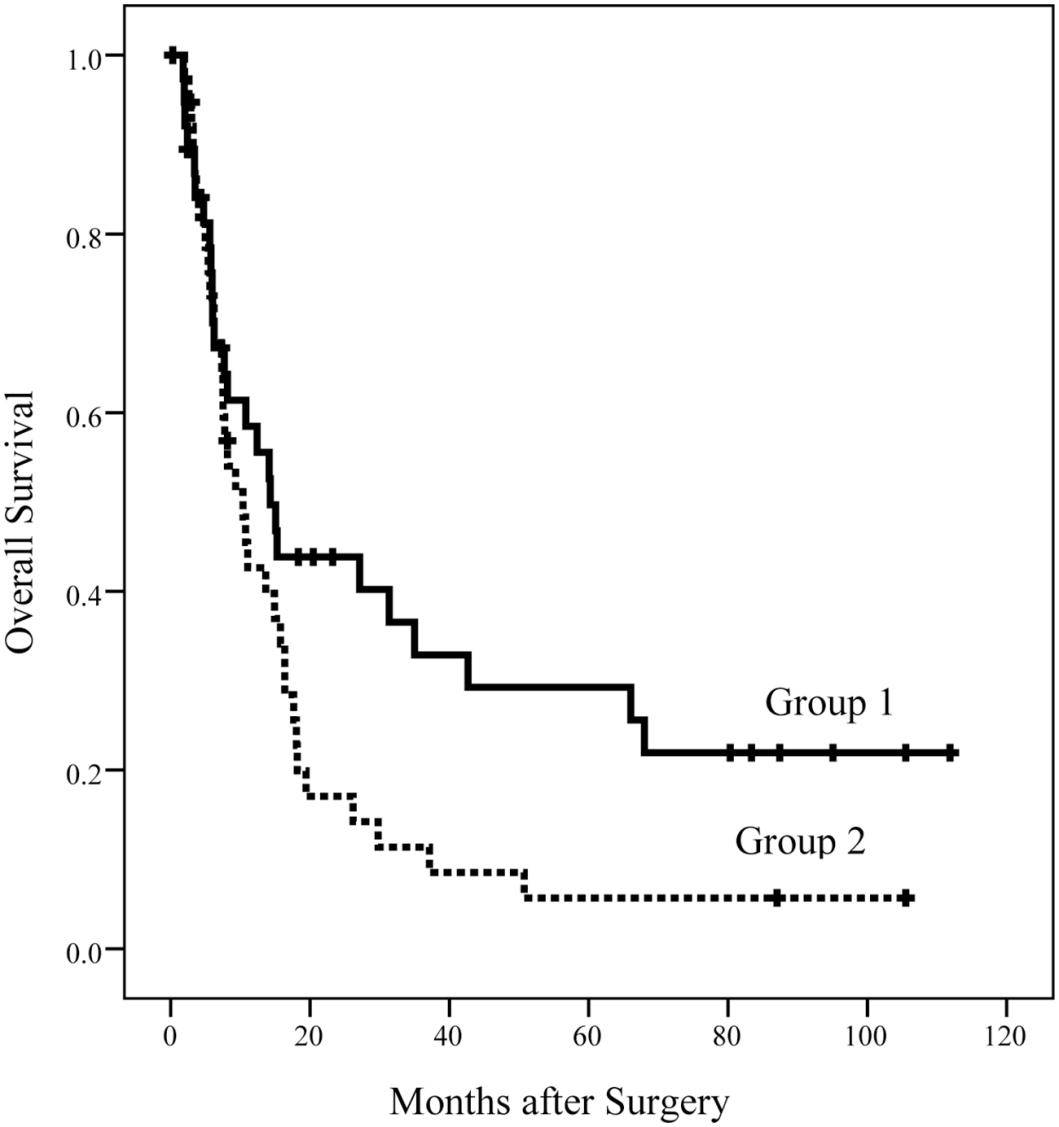
OS of patients in the two groups. The median OS duration in group 1 was significantly longer than in group 2 (14.3 vs. 10.4 months, *p* = 0.047).

**Fig 2:**
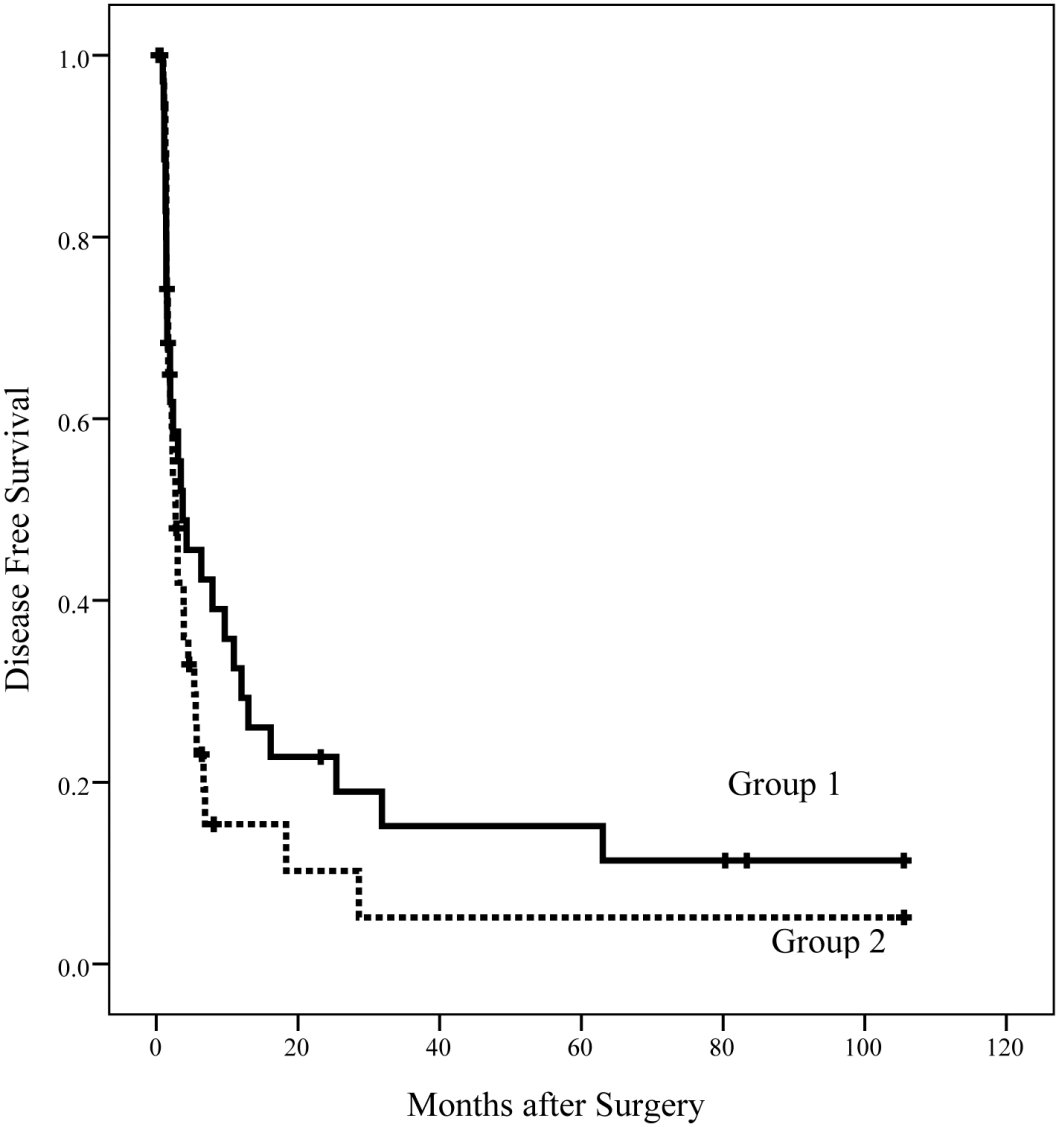
DFS of patients in the two groups. The median DFS duration in group 1 was longer than in group 2 but was not significantly different (3.7 vs. 2.7 months, *p* = 0.191).

Fig 1 shows a Kaplan-Meier plot of the overall survival (OS) of the patients in the two groups. The median OS duration in group 1 was significantly longer than group 2 (14.3 vs. 10.4 months, *p* = 0.047). The 1-, 2-, 3-, and 5-year OS rates in group 1 were 58.5, 43.9, 32.9, and 29.2%, respectively. The 1-, 2-, 3-, and 5-year OS rates in group 2 were 42.6, 17.1, 11.4, and 5.7%, respectively. Fig 2 shows the disease free survival (DFS) of the patients in the two groups. The median DFS duration in group 1 was longer than group 2, but this difference was not significant (3.7 vs. 2.7 months, *p* = 0.191). The 1-, 2-, 3-, and 5-year DFS rates in group 1 were 32.5, 22.8, 15.2, and 15.2%, respectively. The 1-, 2-, 3-, and 5-year DFS rates in group 2 were 15.4, 10.3, 5.1, and 5.1%, respectively.

**Fig 3:**
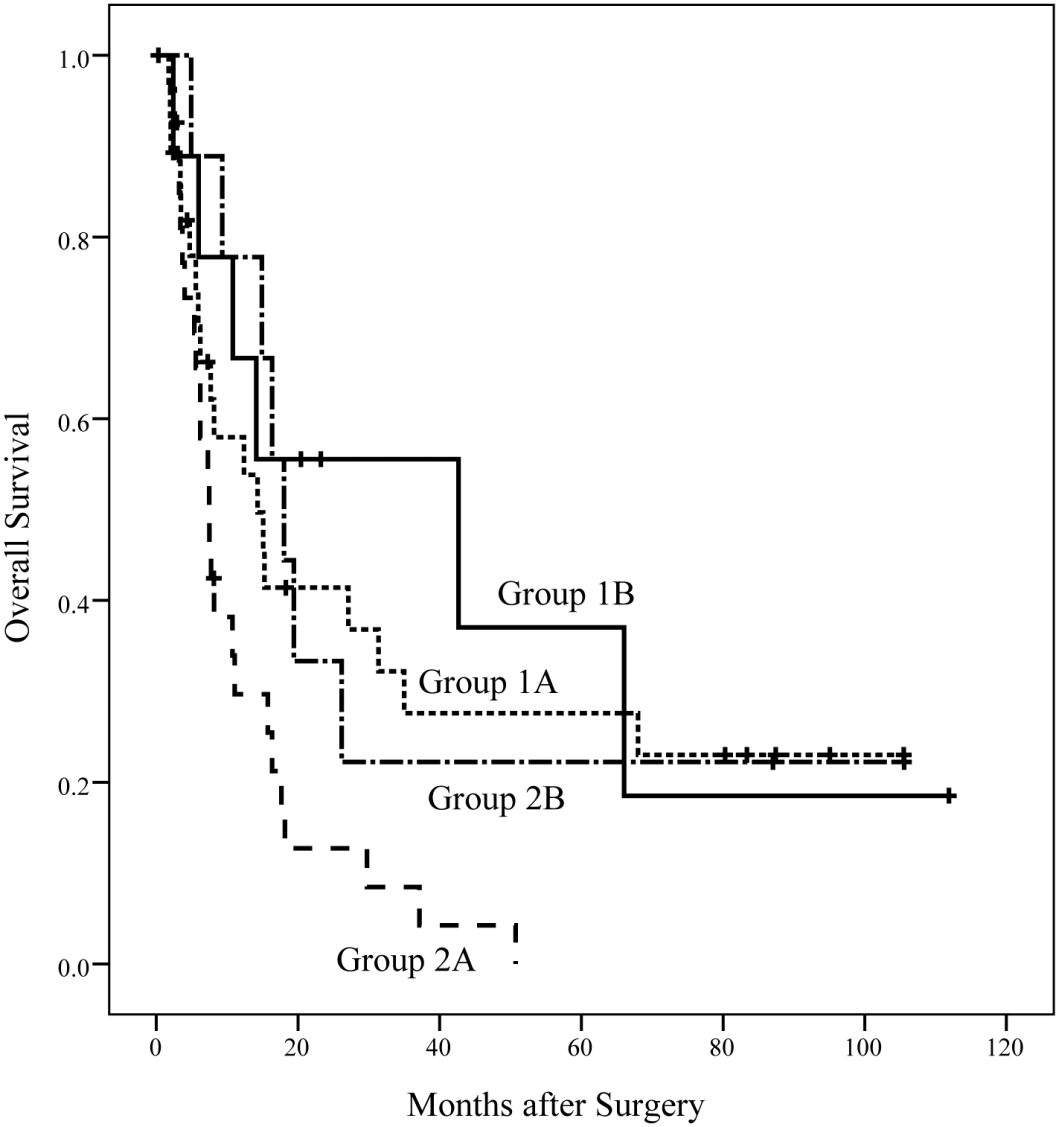
OS of patients in the four subgroups. The median OS durations in groups 1A, 1B, 2A, and 2B were 14.3, 42.7, 7.5, and 18.0 months, respectively, which were significantly different(*p* = 0.018).

**Fig 4:**
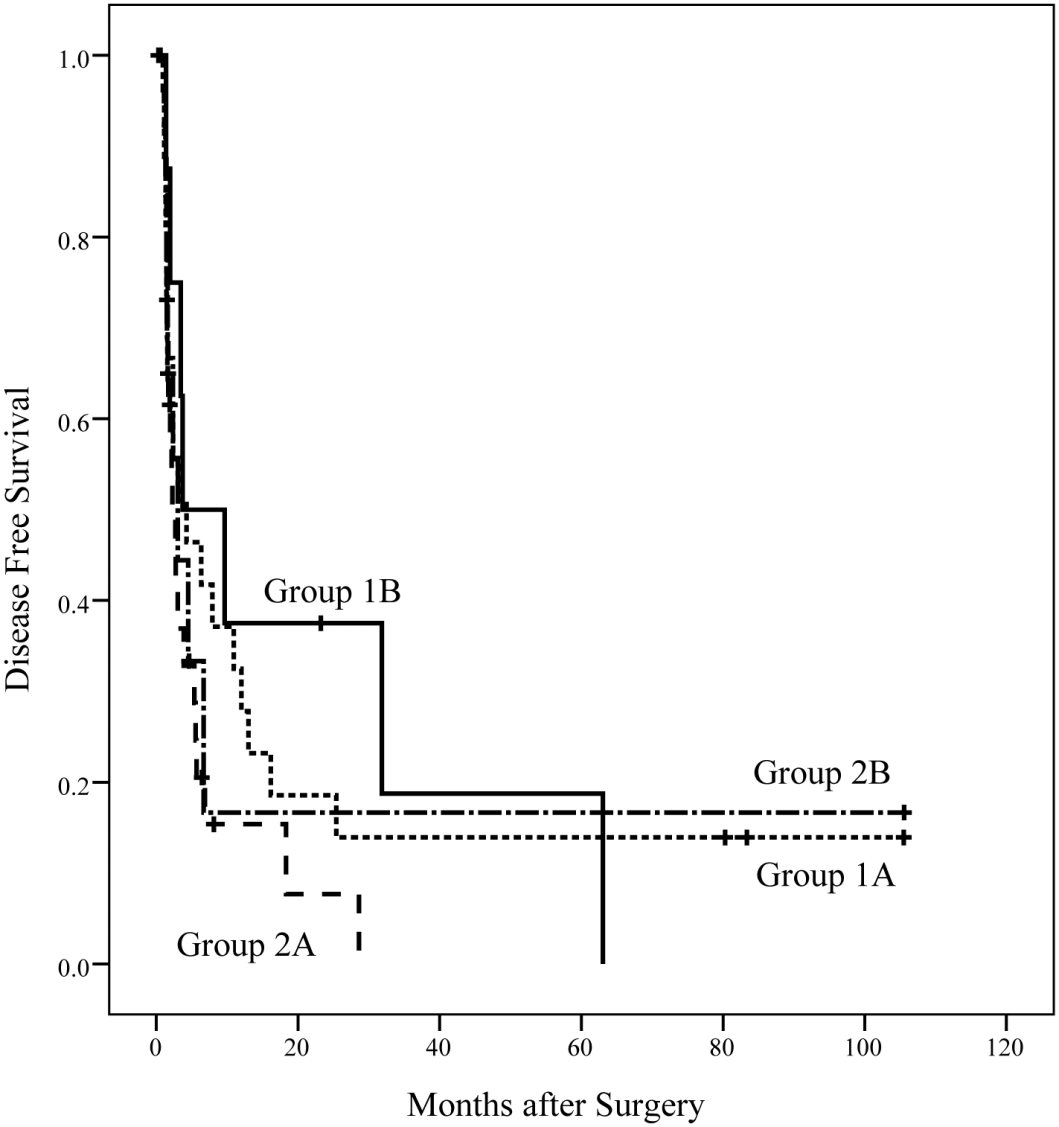
DFS of the patients in the four subgroups. The median DFS durations in groups 1A, 1B, 2A, and 2B were 4.3, 3.7, 2.3, and 3.0 months, respectively, which werenot significantly different (*p* = 0.337).

Fig 3 shows a Kaplan-Meier plot of the OS of the patients in the four subgroups. The median OS duration in groups 1A, 1B, 2A, and 2B were 14.3, 42.7, 7.5, and 18.0 months, respectively, which were significantly different (*p* = 0.018). Fig 4 shows the DFS of patients in the four subgroups. The median DFS duration in group 1A, 1B, 2A, and 2B were 4.3, 3.7, 2.3, and 3.0 months, respectively, which was not significantly different (*p* = 0.337).

### Recurrence patterns and treatments

The patterns of recurrence and postoperative treatments of the patients in the two groups are shown in Table 3. The recurrence patterns were not significantly different between the two groups.

**Table 3:** Patterns of recurrence and postoperative treatments of the patients in the two groups.

	Group 1	Group 2	*p* value
Recurrence	n = 38	n = 39	0.566
Negative	3 (7.9%)	1 (2.6%)	
Positive	28 (73.7%)	31 (79.5%)	
Unknown	7 (18.4%)	7 (17.9%)	
Recurrence Pattern	n = 28	n = 31	0.848
Intrahepatic only	19 (67.8%)	20 (64.5%)	
Extrahepatic only	2 (7.1%)	4 (12.9%)	
Both	7 (25%)	7 (22.5%)	
Treatments			
TACE	21	15	
Hepatectomy	2	0	
PMCT	3	1	
Systemic chemotherapy	1	2	
RFA	2	3	
PEI	1	1	
CIK	2	0	
TAI	1	1	
Sofafenib	1	0	
Sealed source radiotherapy	1	2	
Traditional Chinese medicine	2	2	
Supportive care only	14	18	

Some patients accepted more than one type of treatment

### Multivariate analysis

Table 4 shows the results of a multivariate analysis performed to evaluate the prognostic factors for HCC patients with PVTT/HVTT. Preoperative AFP levels (*p* = 0.011) and the group division described previously (*p* = 0.006) were prognostic factors in HCC patients with PVTT/HVTT. Blood loss and blood transfusion during operation, which were significantly different between the two groups, were not identified as prognostic factors using a Cox’s proportional hazard model (*p* = 0.323 and 0.571, respectively).

**Table 4:** Multivariate analysis results.

	Multivariate analysis		
Variables	Hazard ratio	95% CI	*p* value
Age (≤40 vs. >40 years)	1.626	0.756–3.495	0.165
Preoperative AFP level (≤400 vs. >400 ng/ml)	0.431	0.226–0.822	0.011
Group (1 vs. 2)	0.405	0.213–0.770	0.006
Preoperative ICGR15 (≤10% vs. >10%)	1.534	0.562–4.191	0.292
Preoperative Child-Pugh score (A vs. B)	0.813	0.322–2.049	0.418
Blood loss (≤400 vs. >400 ml)	0.989	0.442–2.211	0.323
Blood transfusion (no vs. yes)	0.955	0.462–1.974	0.571
Maximum diameter of tumor (≤8 vs. >8 cm)	0.780	0.392–1.554	0.358
Number of tumors (solitary vs. multiple)	0.648	0.323–1.297	0.184
Total operation time (≤180 vs. >180 min)	0.970	0.465–2.025	0.806

### Survival analysis of other classification methods

We also conducted a survival analysis of the other classification methods, but all failed to exhibit differences. 1) Considering the extent of the PVTT/HVTT only, independent of the surgeons’ technique during the operation, we divided the patients into two groups: group E1: the PVTT/HVTT involved the segmental or 3^rd^ branches of the portal vein or hepatic vein, and group E2: the PVTT/HVTT involved the 1^st^ branches or the main portal vein trunk/inferior vena cava (*p* = 0.124). 2). If we considered the surgeons’ techniques during the operation independent of the location and extent of the PVTT/HVTT, two types of groups could be established: minor vs. major hepatectomy (as defined previously) and anatomic vs. non-anatomic hepatectomy (*p* = 0.372, and 0.987, respectively). 3) If we considered the surgical margins only, independent of other factors, the patients could be divided into two groups: group M1: surgical margins≤1 cm, and group M2: surgical margins>1 cm (*p* = 0.084). (data not shown in figure or table)

## Discussion

HCC has a high predilection for vascular invasion. Poon et al. reported that PVTT was observed in 64.7% of HCC patients at autopsy [20]. PVTT/HVTT may lead to wide dissemination of the tumor throughout the liver and exacerbate portal hypertension, resulting in liver failure or life-threatening variceal bleeding [6].

There is little consensus regarding the optimum treatment strategy for HCC patients with PVTT/HVTT. TACE is a widely used palliative treatment for these patients. Some researchers have suggested that TACE might be safe and effective for HCC patients with PVTT/HVTT if the patients have good hepatic reserve function and collateral circulation around the portal trunk [21,22]. However, the median survival time with this treatment was only 5.5 to 9.5 months [21–23]. The outcomes of other palliative treatments were discouraging. The median survival time of systemic or regional chemotherapy was 3.9 to 10.2 months [24–27], which could be improved to 11.8 months by including subcutaneous alpha-interferon and chemotherapeutic agents [28]. Transarterial radioembolization with yttrium-90 microspheres or iodine-131-labeled lipiodol had a median survival time of 5.6 to 10.0 months [29–31]. The median survival time with sorafenib therapy was 5.5 to 8.1 months[32,33]. With advances in three-dimensional planning tools, three-dimensional conformal radiotherapy (3-D CRT) allows clinicians to escalate radiotherapy doses to the tumor and minimize radiotherapy doses to normal tissue, such as normal liver parenchyma [6]. Therefore, 3-D CRT has been recently more widely used in cases of HCC with PVTT/HVTT. However, the median survival time was 6.7 to 8.0 months [34,35], which is unsatisfactory.

Resection may be the best and only possibly curative treatment for some HCC patients with PVTT/HVTT. In a number of reports that included all degrees of PVTT/HVTT, the median survival durations varied from 9.0 to 26.0 months [13,15,16,23,36–38], which was even longer in some subgroups with particular clinicopathological and surgical characteristics, such as the thrombus confined to the first or second branch of the main portal vein, prothrombin activity≥75%, maximal tumor diameter <5 cm, and less than three primary nodules [7,15,38,39]. In the present study, the median OS duration of all patients was 12.4 months, and the 1-, 2-, 3-, and 5-year survival rates were 50.4%, 30.1%, 21.8%, and 16.7%, respectively. When the PVTT/HVTT was located in the hepatic resection area and removed with the tumor en bloc (group 1), the median OS duration was 14.3 months. Moreover, in the 9 cases whose surgical margins were >1 cm in group 1 (group 1B), the median OS duration was as long as 42.7 months.

Approximately just 10% of patients with HCC and PV/HVTT can be treated with liver resection, the remaining patients being considered inoperable[7]. The patients who could tolerate surgery generally had relatively lower tumor burden, better liver function reserve, and smaller extent of thrombus. Consequently, we recommend prioritizing resection over other treatments in some patients who meet particular conditions rather than in all patients with HCC and PVTT/HVTT. In addition, we recommend against relying on resection only but suggest the addition of the most appropriate adjuvant therapeutic strategy pre- or/and post-operation based on the clinical and pathological characteristics of each individual patient. The goal of surgeons should be to use a multidisciplinary approach to help patients achieve the longest possible survival duration.

Numerous researchers have adopted a classification similar to these divisions based on the location and extent of the PVTT/HVTT in the present study (group 1 and 2) [7,15–17]. However, few have included surgical margins in the classification. The inclusion of the surgical margins in the classification method (i.e., group 1A, 1B, 2A, and 2B) encourages surgeons to make a conscious effort to widen the surgical margins as much as possible during the operation to prolong patient survival. Surgeons cannot change the location and extent of the thrombus but can widen the surgical margins and remove the thrombus with the tumor en bloc in a considerable number of cases. The results of the present study may encourage surgeons to value the importance of adequate surgical margins and en bloc resection. However, the surgical margins were often close because tumors are large, close to important structures or the future liver remnant may be too small[40,41]. Therefore, we suggested surgeons should widen the surgical margins under the premise of integrity excision of tumor, not damage of uninvolved important structures, and enough remnant liver volume, rather than widen the surgical margins blindly despite concrete conditions.

Notably, although the OS duration of the two classifications was significantly different, the DFS was not significantly different. These results may confirm that tumor recurrence in the remnant liver after surgery was common and nearly inevitable in HCC patients with PVTT/HVTT, which was a major cause of the unsatisfactory prognosis[16,42]. The significant difference in OS duration suggests that survival can be prolonged in some patients by widening the surgical margins and removing the thrombus and tumor en bloc. Further, adjuvant treatment, such as TACE and TAI, after the operation could also significantly improve the prognosis of HCC patients with PVTT/HVTT [42,43]. A better surgical strategy could decrease portal vein pressure and reduce the tumor burden, thus improving liver function, creating conditions for further adjuvant therapy, and producing longer survival [16,44].

The power of the present research was limited by its retrospective nature, single center data, and relatively small sample size. However, the results are valuable for guiding surgical treatment for HCC with PVTT/HVTT. Multicentre prospective registries are required to provide large volumes of patient experience in a timely fashion.

Although only 77 cases were included in our study, achieving this number of cases was difficult given the rarity of HCC patients with PVTT/HVTT who are suitable for hepatic surgery. Moreover, we are the first to introduce surgical margins into the classification system and obtained positive results, which was meaningful for optimizing the surgical treatment strategy in these patients.
